# See-through observation of malaria parasite behaviors in the mosquito vector

**DOI:** 10.1038/s41598-019-38529-3

**Published:** 2019-02-11

**Authors:** Toshiyuki Mori, Makoto Hirai, Toshihiro Mita

**Affiliations:** Department of Molecular and Cellular Parasitology, Juntendo University, 2-1-1 Hongo, Bunkyo, Tokyo Japan

## Abstract

Although it is known that malaria parasites proliferate in the midgut of mosquito vector, their detailed behaviors, from gamete maturation to formation of next generation sporozoite, have not been fully understood at cellular or molecular level. This is mainly attributed to technical difficulties of dissection and whole-mount observation, of delicate and opaque mosquito body contents. In addition, blood pigment surrounding parasites immediately after blood meal also complicates tracing mosquito-stage parasites. Recent revolutionary studies have overcome such negative factors in tissue observation by clearing organisms. CUBIC reagents succeeded to remove both light scattering and blood pigment from various mouse tissues, and to whole-organ image fluorescence-labeled cell structures. In this study, we utilized the advanced version of CUBIC technology and high sensitivity fluorescent markers for see-through observation of mosquito vector after engulfment of rodent malaria parasites to clarify their behaviors during mosquito stage. As a result, we succeeded to visualize oocysts, sporozoites, female gametes and ookinetes in the mosquito bodies without any dissection.

## Introduction

Malaria is one of the three major infectious diseases and brings more than 200 million patients leading to more than 400 thousand deaths, per year worldwide^1^. Malaria symptom is mainly caused by asexually-proliferative parasites in the red blood cells (RBCs) of host patients. A small proportion of such parasites differentiate into male and female gametocytes (gamete precursors) during asexual reproduction^2,3^. Immediately after the gametocytes are engulfed into *Anopheles* mosquito vectors by sucking blood of the patients, they develop into mature gametes and perform sexual reproduction in the midgut^2,3^. The fertilized female gametes are converted to motile ookinetes to migrate outside midgut and produce oocysts, in which they proliferate and differentiate into a number of sporozoites^2,3^. The mature sporozoites egress the oocysts and migrate into salivary grands to wait for next infection to humans. Because only one pair of gamete fusion results in bearing several thousands of sporozoites, the sexual reproduction is one of the most important stages in the life cycle of malaria parasites^2,3^. To date, several studies have tried to understand and attack the molecular mechanism of parasite behaviors in the mosquito stage, to block transmission of malaria disease^4^. Elucidating gamete fusion mechanism is especially highlighted because its prevention is expected to lead to elimination of malaria parasites. Previous studies using knockout parasites and antibodies have identified some factors critical to fertilization^5–8^, in both male and female gametes, and indeed such findings have been applied to development of vaccine targeting proteins crucial to mosquito stage, by which parasite transfer is prevented due to abortion of life cycle in the mosquito vectors^9–12^. To assess the effect of such gene knockout and vaccines, i.e. the function of targeted proteins, establishment of methods to analyze parasite behaviors within the mosquito vector is essential. However, current methods are almost limited to observation aiming to simply confirm presence or absence of parasite population in isolated mosquito organs, such as midguts. Such methods may be useful to assume the developmental stage of defective parasites in the mosquito, but not to analyze detailed phenotypes of individual parasite. On the other hand, electron microscopy of dissected mosquito organs may be effective to analyze detailed phenotypes of parasites, but not to acquire their behaviors as a whole population in a spatially-limited section.

Recently, several studies succeeded to analyze internal cell- and molecular structures in multicellular tissues in mammals and plants, using tissue-clearing technologies; Sca*l*e, CUBIC, ClearSee and TOMEI^13–16^. It is noteworthy that such technologies enable to observe both detailed organelle- and protein complexes within a cell and their distribution throughout the tissue^17–21^. Here, we report a new approach to observe malaria parasite behaviors within mosquito body with no disassembling organs but conserved host-parasite relationships, using advanced CUBIC technology^22^ and high-sensitivity fluorescent marker proteins. This method should be helpful to not only visualize morphological abnormalities in malaria parasites after gene knockout or treatment with transmission blocking vaccine, but also elucidate unknown molecular relationships between parasites and mosquito organs, controlling life cycle of mosquito stage.

## Results

### Clearing mosquito vectors and visualization of GFP-labeled *P*. *berghei*

**Figure 1:**
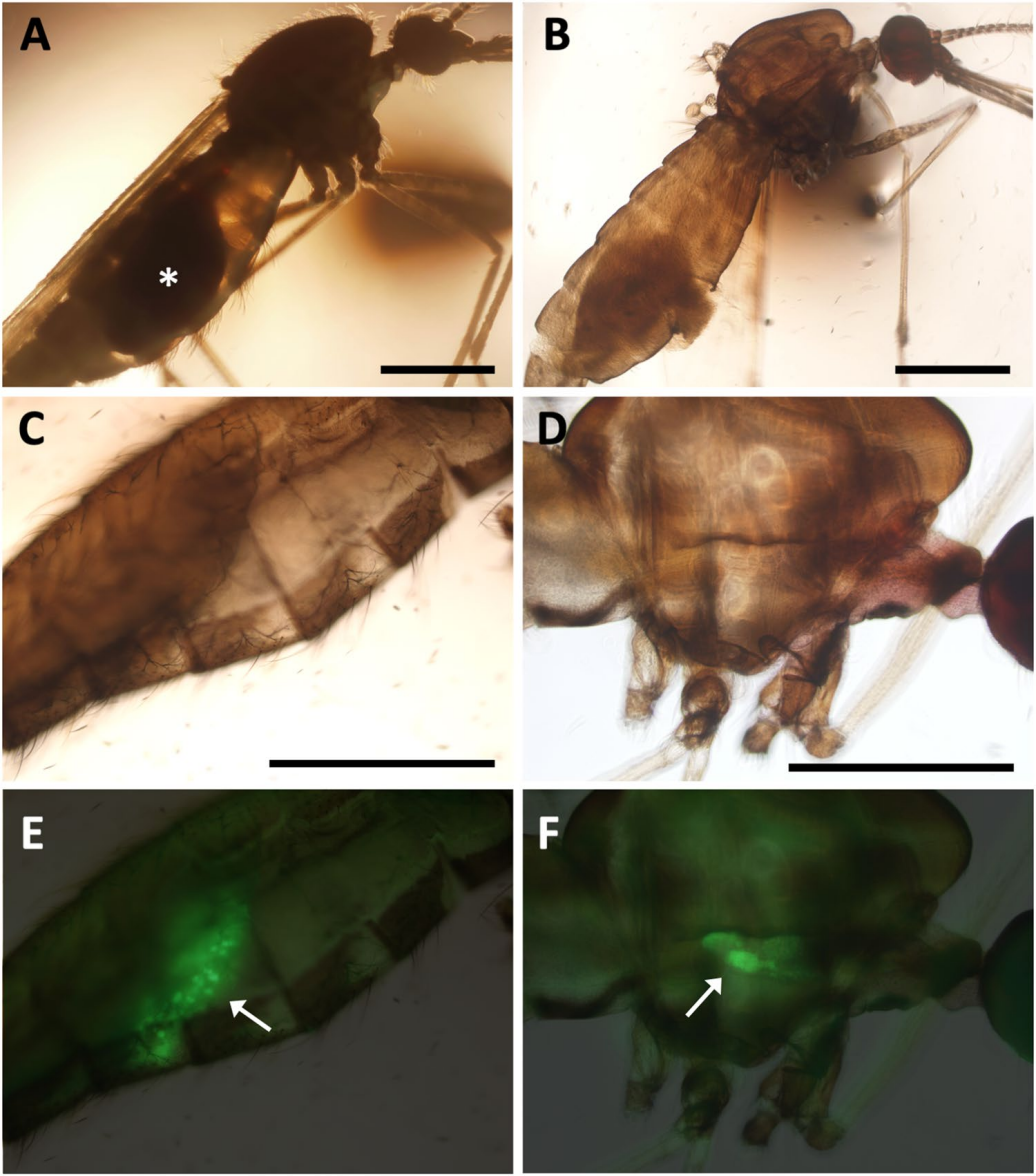
Clearing of mosquitos after malaria parasite ingestion. (**A**) Mosquito before clearing treatment. The midgut is filled with mouse blood (asterisk). (**B**) Transparent mosquito after treatment with CUBIC1 and 2 solutions. (**C**–**F**) Bright field (**C**,**D**) and fluorescence (**E**,**F**) microscopy of transparent stomach (**C**,**E**) and chest (**D**,**F**) regions of mosquito after sacking the malaria parasite strain expressing GFP. The arrows represent sporozoite contained in oocysts (**E**) and salivary grands (**F**). Scale bars represent 1 mm (**A**,**B**); 500 µm (**C**,**D**).

As shown in Fig. 1A, the internal structures of *Anopheles* mosquito body are thoroughly obscure because of strong shadow produced by light scattering, in light microscopy. Especially in the stomach region immediately after blood meal, light absorption by strong blood pigment, i.e. heme, makes it difficult to observe midgut contents (Fig. 1A). Similar light absorption is also observed in fluorescence microscopy (Fig. S1). The original papers of Sca*l*e and the advanced CUBIC technologies have reported that urea and detergent (e.g. Triton X-100) are potentially effective to remove light scattering produced by protein and lipid structures respectively^22^. Indeed, CUBIC reagent-1 containing 25% urea and 15% Triton X-100 proved to reduce light scattering in mosquito body before blood meal (Fig. S2A). In addition, following treatment with CUBIC reagent-2 containing 50% sucrose further increased transparency of mosquito body, matching refractive index (Fig. S2B). When the similar treatments were applied to mosquitoes after blood meal, the transparency of midgut region was also increased, because the aminoalcohols in CUBIC reagent-1 (25% *N*,*N*,*N′*,*N′*-Tetrakis(2-hydroxypropyl)ethylenediamine) and -2 (10% 2,2′,2″-Nitrilotriethanol) can effectively remove heme in blood^22^. We, therefore, decided to utilize the advanced components of CUBIC reagents-1 and 2 to clear mosquitos after sucking mouse blood infected by *P*. *berghei*.

*Anopheles* mosquitoes were fed with mice infected by PbHSP70 promoter::GFP-expressing *P*. *berghei*^23^, which enables to trace all the parasite life stages, and reared for 2 weeks to 1 month. When the stomach region of cleared mosquitoes at ~2 weeks after feeding was observed in standard fluorescence microscopy, a number of oocysts emitting GFP signal were obviously detected (Fig. 1C,E). Similarly, the transparent chest region showed salivary grands containing GFP-labeled sporozoites, in the mosquitoes at 1 month after feeding (Fig. 1D,F). Although a previous study has detected similar GFP signals in both developed oocysts and salivary glands of non-cleared mosquito body^24^, our clearing method further enabled to detect parasites before completion of oocyst development, which were obscure in non-cleared mosquito body (Fig. S3), implying it is also applicable to imaging of individual parasites before oocyst formation. In addition, our method could detect both a pair of obvious lobes of salivary gland and sporozoite distribution within them without any dissection (Fig. S4).

### Production of fluorescence marker *P*. *berghei* line to identify cell types of parasite

**Figure 2:**
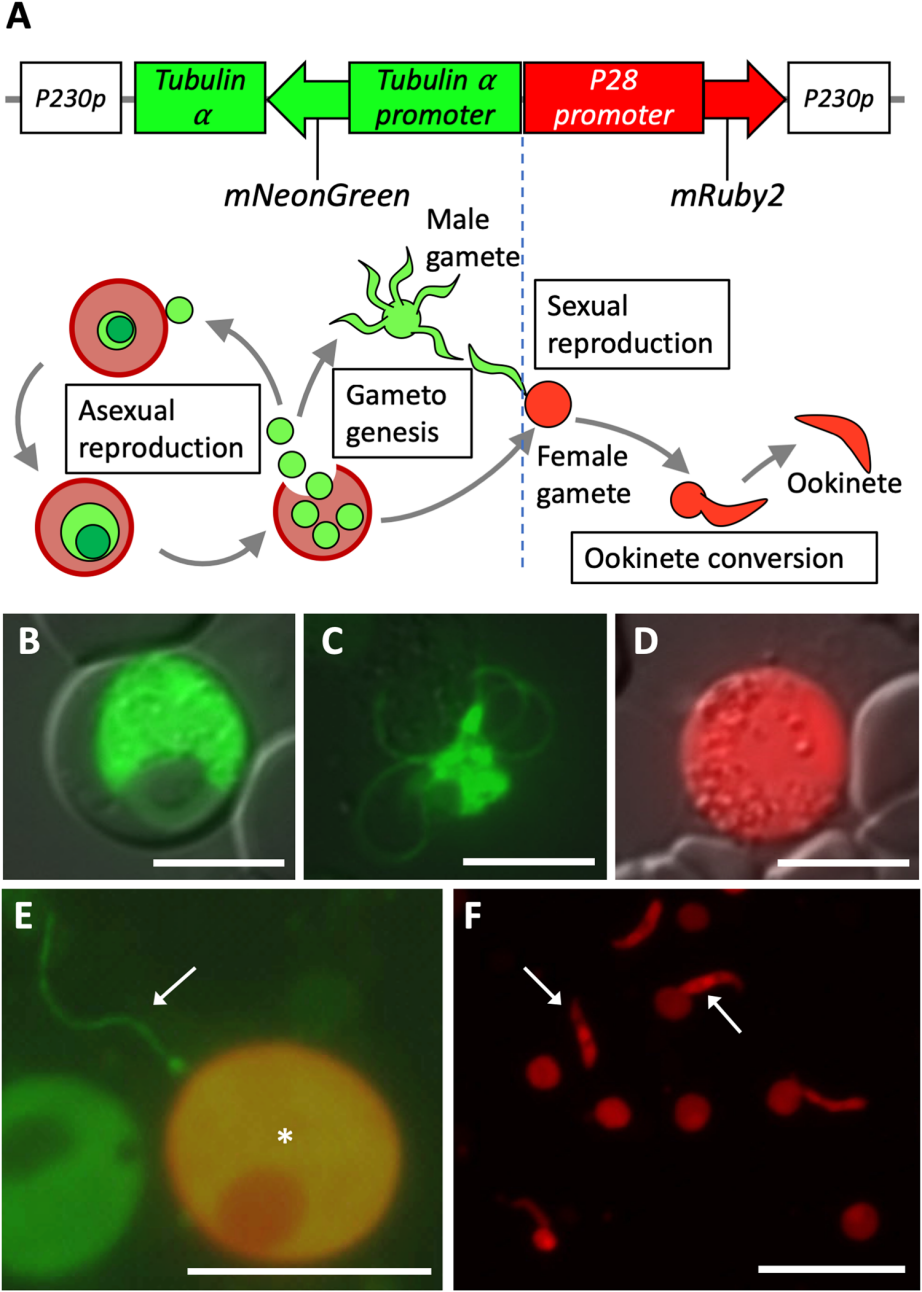
Production of malaria parasite line expressing cell-type specific fluorescent markers. (**A**) Schematic illustration of fluorescent marker genes. They are introduced into *P230p* locus of *P*. *berghei* genome by homologous recombination. The bottom illustration represents *P*. *berghei* life cycle. The colors of parasite cell correspond to fluorescence markers above. (**B**,**C**) mNeonGreen-Tubulin α driven by its own promoter is strongly expressed in asexual stage parasites (**B**) and male gametes (**C**). (**D**) mRuby2 driven by P28 promoter is expressed specifically in female gametes. (**E**) *In vitro* observation of mature gametes. The greenish male (arrow) and reddish female (asterisk) gametes interact each other. (**F**) Ookinete conversion in the fluorescent marker line. The arrows represent mature ookinetes expressing mRuby2. Scale bars represent 10 µm (**B**,**D**,**E**); 5 µm (**C**); 25 µm (**F**).

Because the mosquito stage parasites perform sexual reproduction, in which female gametes convert to ookinetes after fertilization, a fluorescent marker parasite line expressing cell-type specific markers is required to trace gametes, distinguishing them from different-type cells. mNeonGreen and mRuby2 were recently developed as high-sensitivity green- and red fluorescent proteins, respectively^25,26^. We produced a plasmid vector construct containing *P28* promoter-driven *mRuby2*- and *tubulin α* promoter-driven *mNeonGreen-tubulin α* genes to label female gametes and the other cells, respectively (Fig. 2A). After transfection of the *P*. *berghei* with the construct, the drug selection markers were removed by positive- and negative selection of transformants (see the Materials and Methods). Most blood-stage asexual cells and male gametes, of the transformants, strongly expressed mNeonGreen signal (Fig. 2B,C), whereas mRuby2 was expressed specifically in female gametes due to the promoter derived from female specific gene *P28* (Fig. 2D). It is also noteworthy that those markers are almost exclusive to each other and mRuby2-positive female gametes are almost mNeonGreen-negative (Fig. S5), helping to clearly distinguish them from asexual cells and male gametes. We named the double marker line, 28R/GTA, in this study. When *in vitro* fertilization assay was performed, gamete interaction was frequently detected between mNeonGreen-labeled male and mRuby2-labeled female (Fig. 2E). In addition, we also succeeded in live cell imaging of mature male gametes using the same marker line (Movie S1), suggesting that it is also available for real-time imaging of *in vitro* sexual reproduction of *P*. *berghei* in future. After overnight incubation of fertilized female gametes, a number of ookinetes emitting mRuby2 signal were observed (Fig. 2F), confirming that the fluorescent labeling did not affect their fertility at all and implying that the change of signal shape involving ookinete formation can be a marker of successful fertilization in the mosquito body.

### See-through observation of ookinete conversion in the transparent mosquitoes

**Figure 3:**
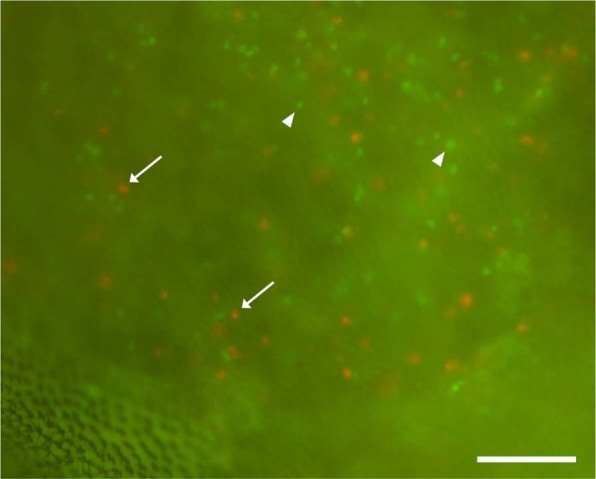
Epi-fluorescence microscopy of cleared mosquito stomach containing fluorescent marker parasites. Arrows and arrow heads represent examples of female gametes expressing mRuby2 and asexual parasites or male gametes, expressing mNeonGreen, respectively. Scale bar represents 50 μm.

**Figure 4:**
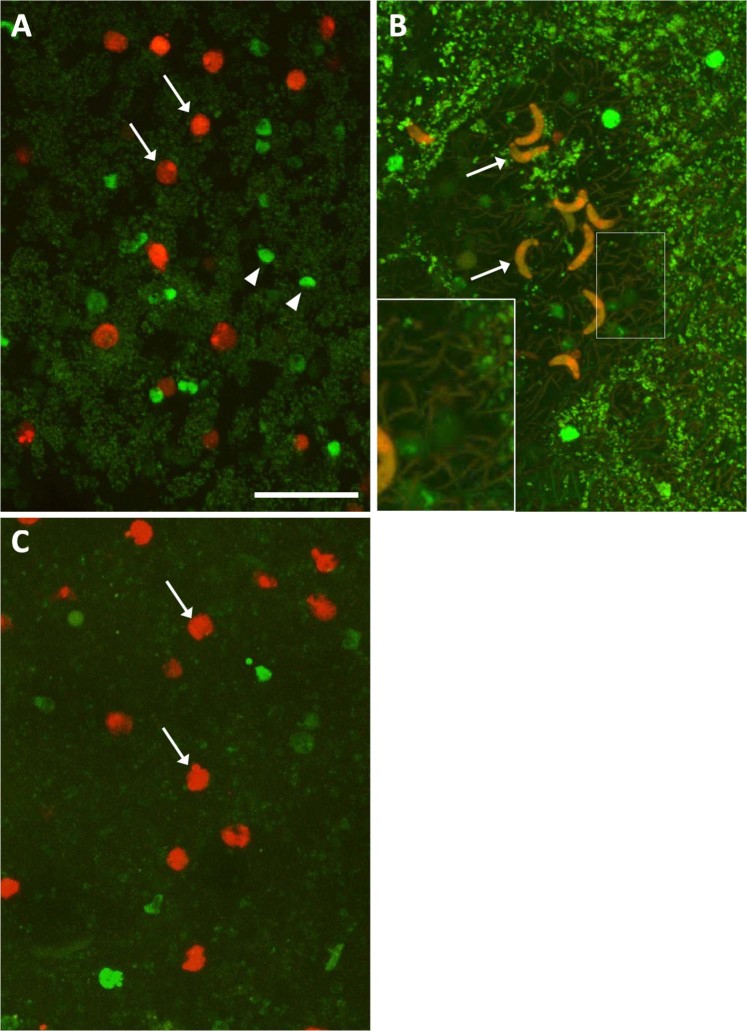
Confocal microscopy of gamete behaviors in the transparent mosquito midgut. (**A**) A number of female gametes expressing mRuby2 (arrows) are detected in the midgut of mosquitos at 10 min after sacking mouse blood infected with the fluorescent marker line. The greenish signals are from asexual cells and male gametes, expressing mNeonGreen-tubulin α (arrowheads). (**B**) Most female gametes are converted into ookinetes (arrows) at 22 h after ingestion of similarly-infected blood. The insert represents a high-magnification of the boxed region showing microvilli. (**C**) No ookinete conversion occurs in GCS1 KO female gametes (arrows) at 22 h after ingestion. Scale bar represents 30 µm.

Since the CUBIC 1- and 2 reagents proved to be capable of clearing mosquito midgut, we performed see-through observation of the 28R/GTA parasites in the mosquito midgut. At 10 min after feeding, the mosquito vectors containing 28R/GTA parasites were fixed and cleared. When the stomach region was observed in an epi-fluorescence microscopy, a number of red and green fluorescent signals were clearly detected as a marker of female gametes and the other cell-type parasites (i.e. asexual cells and male gametes), respectively (Fig. 3). To remove defocused fluorescent signals and autofluorescence from mosquito body structures, confocal microscopy was performed, using same samples as above. As a result, the parasite cells were further obviously detected (Fig. 4A). Unfortunately, no mature male gametes were observed probably because of relatively weaker and thinner signal. When the mosquitoes at 22 h after feeding were similarly observed, almost all female gametes were converted into ookinetes (Fig. 4B), indicating that their fertilization and zygote formation are completed in the midgut within 22 h. Microvilli surrounding ookinetes were occasionally detected in a surface part of midgut, due to their autofluorescence (Fig. 4B, insert). To validate an availability for analysis of sexual reproduction in the midgut, we disrupted *GCS1* gene, which was identified as a gamete fusion factor expressed exclusively in the male gamete^5,6^, and performed similar observation on the GCS1-knockout 28R/GTA line. As a result, no ookinete conversion was observed in the female gametes even at 22 h after feeding probably due to unsuccessful fertilization (Fig. 4C).

## Discussion

In this study, we for the first time succeeded to establish a see-through observation method to trace malaria parasites in the mosquito stage, from ookinete conversion to sporozoite formation in oocyst and its accumulation into salivary glands, by clearing mosquito vectors. Recently, it was reported that the terrestrial isopod *Armadillidium vulgae* and the marine crab *Philyra* sp. are successfully cleared with pigment-bleaching and/or decalcification, followed by the advanced CUBIC method^21^. Although the same study succeeded to visualize internal organs and nuclear distribution by propidium iodide staining of completely-cleared *A*. *vulgae* after the method above, it is yet unknown whether fluorescent protein markers, such as GFP, could be affected by pigment-bleaching and/or decalcification or not^21^. In contrast, our method provided the sufficient clearing condition for the observation of relatively larger structures such as oocysts and salivary glands containing GFP. In addition, the use of confocal microscopy is more effective for detection of smaller cells, such as female gametes and the other stage cells, i.e. asexual cells and male gametes, in the midgut. The mRuby2 driven by *P28* promoter was found to show a strong signal intensity in both the female gametes and ookinetes of the 28R/GTA line, demonstrating that the newly-used red fluorescent marker is useful to sensitively label *Plasmodium* parasites. On the other hand, defective ookinete conversion of GCS1 knockout 28R/GTA line was clearly shown in the mosquito midgut. Although mature male gametes and post-ookinete stage parasites have not yet been successfully observed in the present study due to weak signal of mNeonGreen-tubulin α driven by the *tubulin α* promoter, it may be still possible to produce a construct composed of multiple fluorescent markers, each of which emits different color fluorescence by stage specific promoters, to distinguish all the cell types of parasite in the cleared mosquito body, in future. Furthermore, the drug selection-marker free parasites, such as 28R/GTA line, are also helpful to directly use for gene knockout followed by drug selection. As previous studies have succeeded to visualize and trace migration of sporozoites in the hemocoel just beneath cuticle of live mosquitoes using micromanipulation techniques^27,28^, it would be interesting to observe living gametes during sexual reproduction in the live mosquito midguts. To this end, however, we need to overcome several technical difficulties involving artificial injection of purified gametocytes into the midgut and imaging of vigorously-moving male gametes in the light-scattering midgut structures.

To date, several studies have shown that defective mosquito stages of malaria parasites, which are induced by parasite gene knockout and transmission-blocking vaccine treatments of infected mammalian hosts, lead to arrest of parasite life cycle^5–12^. Of those, the studies on ookinete surface proteins P25 and P28 have shown interesting results, in which antibodies against both proteins critically block transmission, but their double knockout parasites occasionally show both normal and abnormal behaviors during migration across the midgut epithelium^29,30^. Despites several hypotheses proposed to interpret the difficult results, the precise molecular P25/P28 function and the true blocking effect of antibodies against them are still elusive^31^. This may be attributed to observations biased to focusing on individual ookinete behavior. As previous see-through methods based on transparent tissues and fluorescent markers have enabled to observe both whole and individual cell behaviors within a tissue, it may be possible that our present method elucidates any unknown parasite behaviors during the mosquito stage.

## Methods

### Primer sequences

P28promF; TCTCGATATCCGCCGGGGCCCAGCTTTATAGTTATATTTTTGTGG

P28promR; GCCCTTAGACACCATTTTCGTGAAAATTTAATATAAAATAATTG

mRubyF; ATGGTGTCTAAGGGCGAAGA

mRubyR; TGAGCCGTTTAAACTTACTTGTACAGCTCGTCCA

pP28F(IF); CTGTTTAAATATGATATCCGCCGGGGCCCAGCTTTATAG

mRubyR(IF); GTTCATGTACTTGTTTTTACTTGTACAGCTCGTCCA

ptubAF; TGATGGCACGTGGAGATTTATAAGCTTAATGAAAAAGG

ptubAR; CCCTTTGATACCATTTTCGAATAAATTTATCTAAAATAG

TUBAF; TATATAGGTCCGGACTCAGATCTCGAGTGAGAGAAGTTATTAG

TUBAR; GCGGCCGCTTATTCATATCCTTCATCTTC

ptubAF(IF); TGGGCCCCGGCGGATGAGATTTATAAGCTTAATGAAAAAGG

TUBAR(IF); TCTAGAGTCGCGGCCTTATTCATATCCTTCATCTTC.

### Production of a transgenic P. berghei expressing fluorescence markers

The deduced promoter region of P28 gene (PBANKA_0514900) was amplified with primers P28promF and P28promR, and genomic *P*. *berghei* DNA. The mRuby2 cDNA was amplified with primers mRubyF and mRubyR, and pcDNA3.1-Clover-mRuby2 plasmid DNA (addgene). The resultant PCR products of P28 promoter and mRuby2 were conjugated by fusion PCR based on their overlapped sequence^32^, to produce the female specific marker cassette pP28::mRuby2. The cassette was further amplified with primers pP28F(IF) and mRubyR(IF), and the resultant PCR product was cloned into the pL1186 plasmid DNA^33^ after digestion with *Eco*RV and *Pme*I, to exchange the preexisting *5′pblccl::RFP* to the *P28promoter::mRuby2*, using In-Fusion® HD cloning kit (Clontech). The deduced promoter region and coding region of *P*. *berghei tubulin α* gene (PBANKA_0522700) were amplified with pairs of primers ptubAF/ptubAR and TUBAF/TUBAR, respectively. The resultant PCR products and a codon-modified *mNeonGreen* cDNA (GENEWIZ) were fused similarly as described above, to produce the *tubulin α* promoter*::mNeonGreen-tubulin α* cassette. The cassette was further amplified with primers ptubAF(IF) and TUBAR(IF), and the resultant PCR product was cloned into the *P28* promoter*::mRuby2*-containing pL1186 vector (see above) after digestion with *Eco*RV and *Not*I, to exchange the preexisting *PB000791*.*03*.*0::EGFP* to the *tubulin α* promoter*::mNeonGreen-tubulin α* cassette, using In-Fusion® HD cloning kit. The resultant plasmid vector containing both *P28* promoter*::mRuby2* and *tubulin α* promoter*::mNeonGreen-tubulin α* cassettes was designated 28R/GTA plasmid vector.

The rodent malaria parasite *P*. *berghei* ANKA was transfected with the 28R/GTA plasmid linearized after digestion with *Sac*II, by double cross-over homologous recombination at the non-essential *p230p* gene of parasite genome. The transformants were injected into mice (*M*. *musculus* ddY strain) and positively-selected, giving pyrimethamine-containing water to the infected mice^33^. The selected transformants were further negatively-selected, similarly giving 5-fluorocytosine-containing water, so that drug selection marker-free transformants are finally produced^33^. The cloned transformant after limiting dilution was designated 28R/GTA strain.

### *In vitro* fertilization assay

*In vitro* fertilization assay was performed as previously reported^5^. 10 μl of blood from mouse infected with 28R/GTA strain was diluted and incubated in a gametogenesis inducing medium for 15 min or overnight, at 21 °C. The precipitated cells in the medium were recovered after incubation, and observed using a fluorescence microscope (AxioImager M2, Zeiss). The movement of mature male gametes was captured and recorded using a high-sensitivity CCD camera Zyla 4.2 PLUS (Andor).

### Feeding mosquitoes with infected mouse

A mouse infected with *P*. *berghei*, after pentobarbital treatment, was put on a nylon mesh cage containing mature mosquitoes (*A*. *stephensi*) to feed them. The mosquitoes were collected at 10 min or 22 h after feeding, for imaging of 28R/GTA parasites.

### Clearing mosquitoes

The collected mosquitoes after blood meal ingestion were fixed in 4% paraformaldehyde in PBS overnight. The fixed whole mosquito bodies were washed in 0.01% Triton X-100 in PBS for 30 min, followed by washing in DW for 10 min. The washed mosquito bodies were treated with a solution containing 1 mM CuSO_4_ and 50 mM ammonium acetate (pH5.0) for 1 h to suppress autofluorescence from erythrocytes, followed by rinsing in DW for 10 min. The washed whole mosquito bodies were thrown into 1/2 × CUBIC reagent-1 and incubated for 1 h at 37 °C, followed by incubation in 1 × CUBIC reagent-1 for 6 h at 37 °C. The mosquito bodies were washed in 0.01% Triton X-100 in PBS overnight. The washed whole mosquito bodies were thrown into 1/2 × CUBIC reagent-2 and incubated for more than 6 h at 37 °C, followed by incubation in 1 × CUBIC reagent-2 until they become transparent at 37 °C. The components of CUBIC reagent-1 (25% urea, 25% *N*,*N*,*N′*,*N′*-Tetrakis(2-hydroxypropyl)ethylenediamine, 15% Triton X-100) and -2 (50% sucrose, 25% urea, 10% 2,2′,2″-Nitrilotriethanol, 0.1% Triton X-100) were as previously reported^22^. The transparent whole mosquito bodies were observed through cuticle using fluorescence microscopes IX71 (Olympus) and AxioImager M2 (ZEISS), or a confocal laser scanning microscope FV1000-D (Olympus).

### Ethical Approval

Studies with experimental animals were approved by Animal Care and Use Committee of Juntendo University, and followed guidelines of this committee. The transgenic *P*. *berghei* was generated under the guidelines of the recombinant DNA experiments committee of Juntendo University. The assigned ID for above experiments is 25–115.

## Supplementary information

Supplemental Information

Movie S1
