# Supplementary material for: See-through observation of malaria parasite behaviors in the mosquito vector
Source: Sci Rep. 2019 Feb 11;9:1768. doi: 10.1038/s41598-019-38529-3 (PMC6370880; doi:10.1038/s41598-019-38529-3)
Supplement: Supplementary file 1 — Supplemental Information [file 41598_2019_38529_MOESM1_ESM.pdf]

Supplemental Information

See-through observation of malaria parasite behaviors in the mosquito vector

Toshiyuki Mori, Makoto Hirai, Toshihiro Mita
Department of Molecular and Cellular Parasitology,
Juntendo University, 2-1-1 Hongo, Bunkyo, Tokyo, Japan

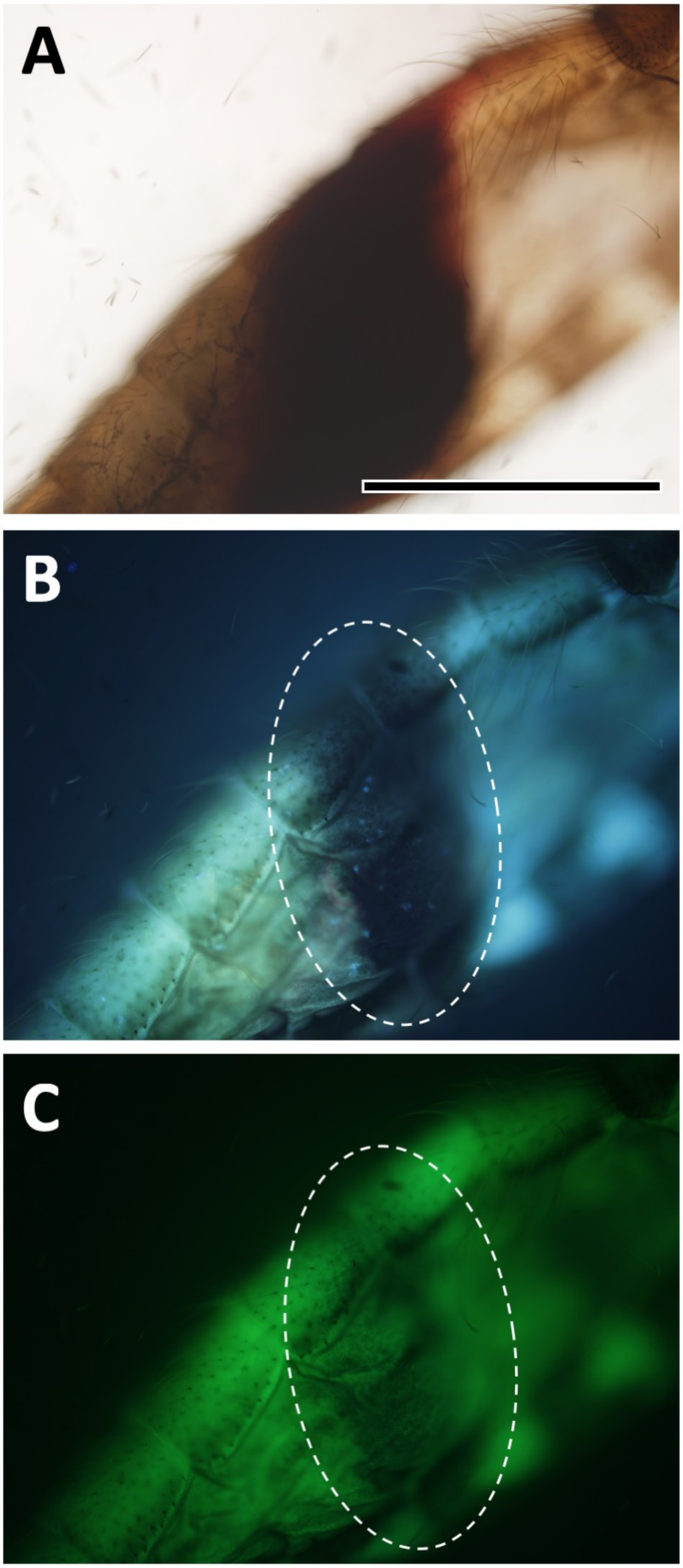

Figure S1. Light microscopy of stomach region of mosquito vector **A**, Bright field image of stomach containing blood meal. The blood pigment makes a strong shadow. **B** and **C**, fluorescence images of the same field as **A**. Both UV- (**B**) and blue (**C**) exciting lights are absorbed by the blood pigment. Scale bar represents 500 µm.

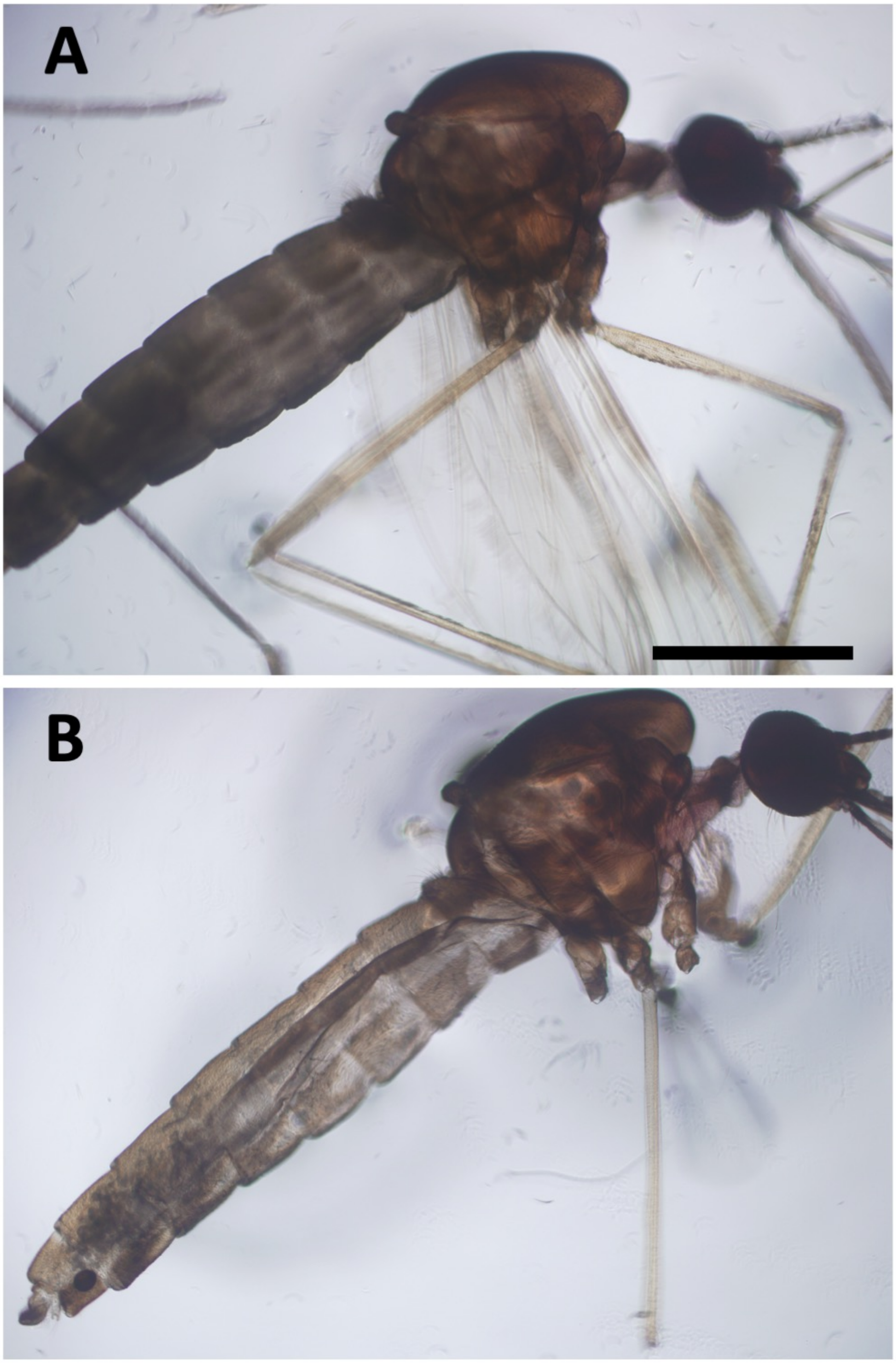

Figure S2. Increase of transparency by treatment with CUBIC regents 1 and 2. **A**, The mosquito body was somewhat cleared by regent 1 treatment alone. **B**, Following treatment with regent 2 increased the transparency. Scale bar represents 1 mm.

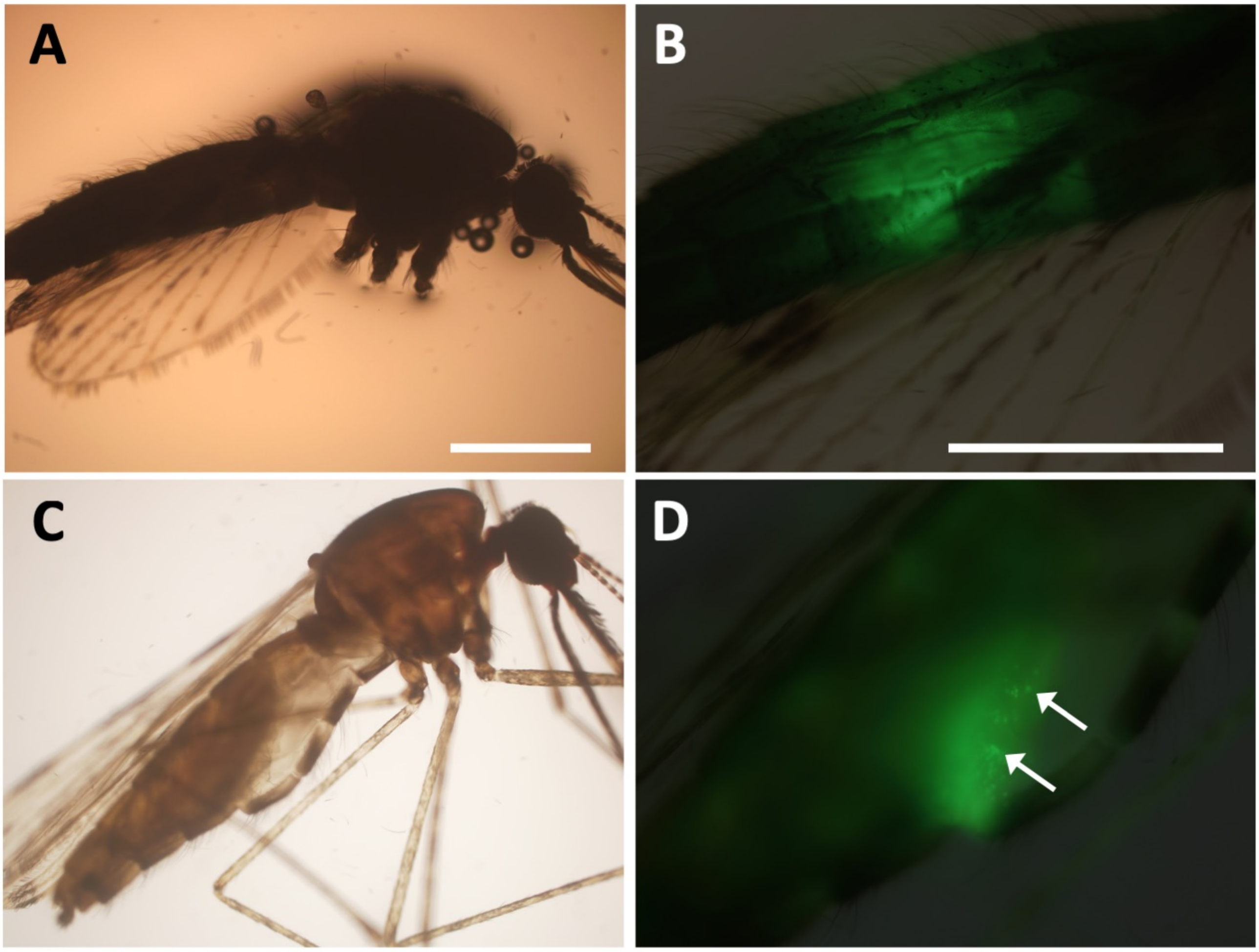

Figure S3. Fluorescence microscopy comparing between non-cleared and cleared mosquito vectors **A**, Bright field image of non-cleared mosquito after ingestion of GFP-expressing parasites. **B** Fluorescence image of the same sample as **A**. **C**, Bright field image of similar mosquito after clearing treatment. **D,** Fluorescence image of the same sample as **C**. The arrows represent examples of parasite before completion of oocyst development, which were obscure in the non-cleared mosquito (**B**). Scale bars represent 1 mm (**A**) and 500 µm (**B**).

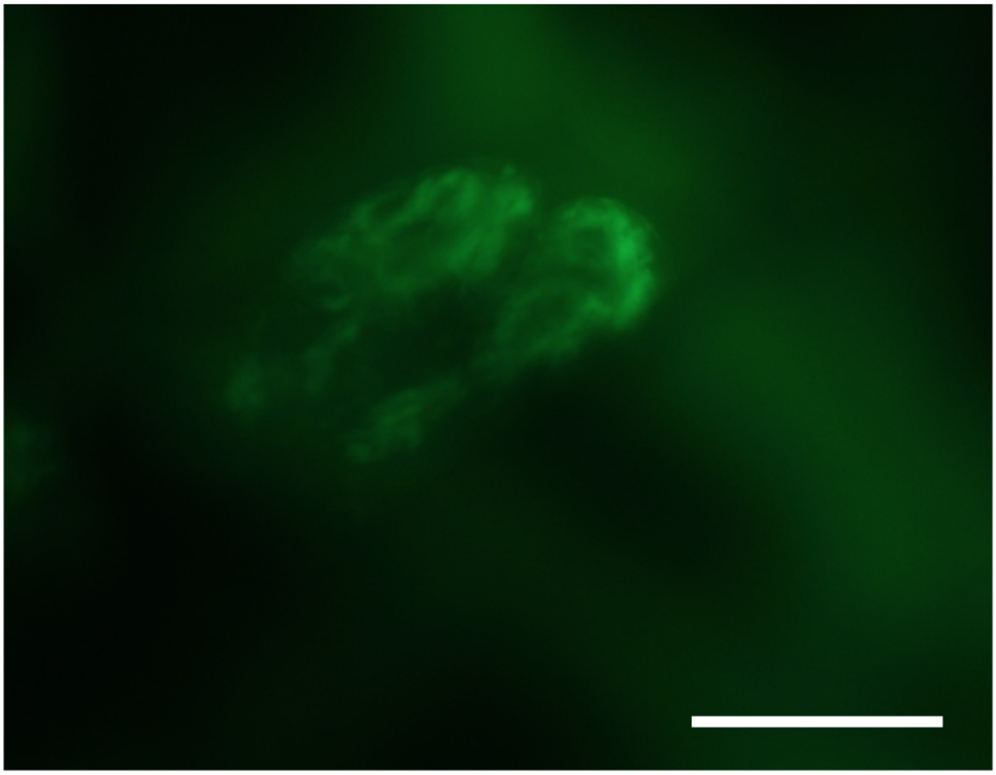

Figure S4. Salivary grands containing GFP-expressing sporozoites. Scale bar represents 100 µm.

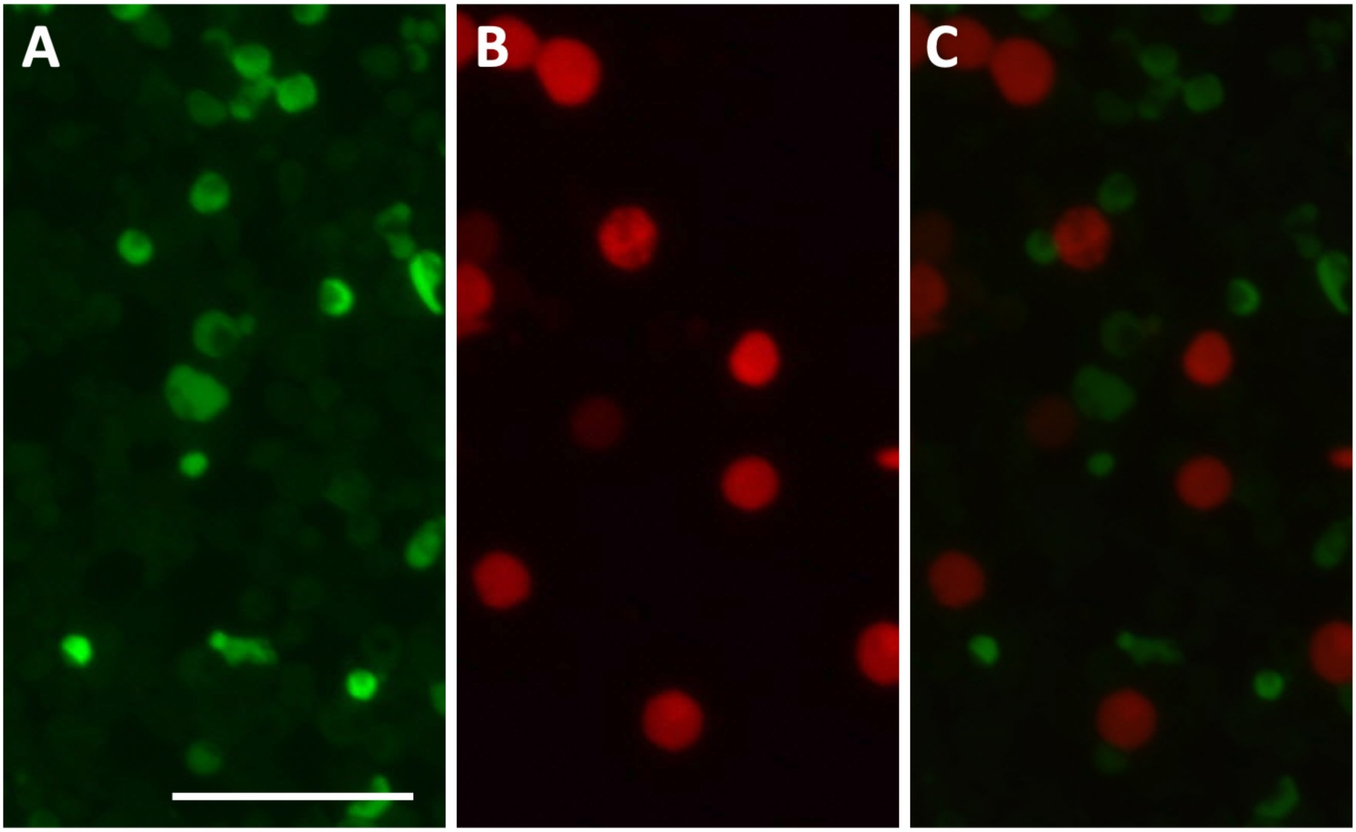

Figure S5. Exclusive expression of mNeonGreen and mRuby2 in the 28R/GTA line. **A** and **B**, Asexual cells and male gametes, both of which express mNeonGreen (**A**), and mRuby2-expressing female gametes (**B**), are detected in an identical field of epi-fluorescence microscopy. **C**, Merge of **A** and **B** shows that almost all female gametes are mNeonGreen-negative. Scale bar represents 25 µm.
